# Appetitive traits and food intake patterns in early life^1^

**DOI:** 10.3945/ajcn.115.117382

**Published:** 2015-12-16

**Authors:** Hayley Syrad, Laura Johnson, Jane Wardle, Clare H Llewellyn

**Affiliations:** 2 Health Behaviour Research Centre, Department of Epidemiology and Public Health, University College London, London, United Kingdom; and; 3 Center for Exercise, Nutrition and Health Sciences, School of Policy Studies, University of Bristol, Bristol, United Kingdom

**Keywords:** appetite, children, food intake, meal frequency, meal size

## Abstract

**Background:** High food responsiveness (FR) and low satiety responsiveness (SR) are 2 appetitive traits that have been associated longitudinally with risk of excessive weight gain; however, to our knowledge, no studies have examined the associations between these traits and eating patterns in daily life in young children.

**Objective:** We tested the hypothesis that higher FR is independently associated with a higher meal frequency and that lower SR is associated with a larger meal size.

**Design:** Data were from 1102 families (2203 children) from the Gemini twin birth cohort. Appetite was assessed with the use of the Child Eating Behavior Questionnaire when the children were 16 mo old (mean ± SD: 15.73 ± 1.08 mo old), and meal frequency (eating occasions per day) and meal size (kilojoules per eating occasion) were determined from 3-d diet diaries completed by parents when the children were 21 mo old (mean ± SD: 20.65 ± 1.10 mo old). Complex samples general linear models were used to explore cross-sectional associations between appetitive traits and meal variables.

**Results:** After adjustment for the covariates gestational age, birth weight, sex, difference in age at diet-diary completion, and appetite measurement, higher FR was associated with more-frequent meals (*B* ± SE: 0.13 ± 0.04; *P* = 0.001) but not with meal size (*P* = 0.41), and lower SR was associated with a larger meal size (*B* ± SE: −47.61 ± 8.79; *P* < 0.001) but not with meal frequency (*P* = 0.15).

**Conclusions:** FR and SR predict different eating variables with more food-responsive children eating more frequently, whereas less–satiety-responsive children eat more food on each eating occasion. Different strategies may be required to reduce the potential effects of FR and SR on weight gain.

## INTRODUCTION

The behavioral susceptibility theory of obesity proposes that individuals who are more responsive to external food cues or less responsive to internal cues for satiety are at increased risk of obesity (1). Behavioral studies in pediatric samples have shown that heavier children consume relatively more food than their leaner counterparts do if food is highly palatable, implicating a heightened responsiveness to external food cues, and less downregulation of subsequent food intake after a food preload, implicating blunted sensitivity to internal satiety mechanisms (2–7), which indicate that individual differences in appetitive traits are present from early life.

The development of the Child Eating Behavior Questionnaire (CEBQ)^4^ (8) and the Baby Eating Behavior Questionnaire (9),which is the infant version, has made it possible to measure several appetitive traits including food responsiveness (FR) and satiety responsiveness (SR) in large population-based samples. Both questionnaires use a parent-report format, which, therefore, benefit from the fact that parents are potentially good informants of their children’s appetitive tendencies across multiple situations (10). Downsides are the subjectivity as well as the scope for bias, but the CEBQ has been validated with the use of directly observed measures of eating behavior (11) as well as having been shown to be stable over time (10). Studies that have used the CEBQ and the Baby Eating Behavior Questionnaire have shown individual differences in SR and FR even in the early post-natal months before solid food is introduced (9). SR and FR have been shown to be related to weight quantitatively across the whole distribution, not simply distinguishing normal weight from obese (1, 11–17), and have been shown to predict weight gain from early in life independent of the baseline weight (13, 19). Within the Gemini study, it was previously demonstrated that, during infancy, the pathway between appetite at 3 mo of age and weight at 15 mo of age is stronger than that between weight at 3 mo of age and subsequent appetite at 15 mo of age (15). However, to our knowledge, no previous study has examined how SR and FR could potentially lead to weight gain in terms of how food is eaten in everyday life (19). Weight gain might be possible by eating too many times during the day (the number of eating occasions) (20) and eating too much each time (energy intake at each eating occasion) (21). In the experimental literature, SR is primarily an “eating offset” trait whereby individuals who have low SR tend to continue eating for longer at a meal (11). Therefore, we hypothesized that this trait would be associated primarily with higher intake at each eating occasion. FR is not as straightforward because food cues can serve both to initiate eating and to prolong a meal if palatable food continues to be offered; however, FR appears to be less-strongly associated with energy intake at a meal than is SR (11); thus, we hypothesized that FR was more aligned to being an eating onset trait and would be associated primarily with eating frequency. We tested these hypotheses with the use of CEBQ data and information on the eating frequency and energy intake per eating occasion in a large sample of 21-mo-old children whose parents had completed 3-d diet diaries.

## METHODS

### Study population

The study population was the Gemini cohort, which was a longitudinal cohort of families with twins born between March and December 2007 (22). All families with live twin births during March to December 2007 in England and Wales (*n* = 6754) were asked by the Office for National Statistics if they would be willing to be sent a research invitation from the Gemini research team; 3435 families agreed, and this invitation was sent on 16 January 2008. A total of 2402 families completed the baseline questionnaire (70% of those willing to be approached). The Gemini sample is representative of United Kingdom twins on the basis of national twin statistics on sex, gestational age, and birth weight (23).

When the twins were ∼16 mo old (mean ± SD: 15.73 ± 1.08 mo old), parents (almost always mothers) completed the CEBQ for each child. When the twins were 21 mo of age (20.65 ± 1.10 mo of age), parents were sent pediatric diet diaries to be completed over 3 d (2 weekdays and one weekend day). Over one- half the baseline sample completed a diary for each child (1357 families; 56.5%), with full 3-d data available for 2336 children. Only children for whom there were also data on the CEBQ, ages at diary and CEBQ completion, birth weight, and gestational age were included. The final sample for analysis was 2203 children because one child in a twin pair was missing birth weight. Parents provided written informed consent for children’s participation, and the study was approved by the University College London Committee for the Ethics of Non-National Health Service Human Research.

### Measures

#### Appetitive traits

The CEBQ is a parent-reported psychometric measure of pediatric appetitive traits (8). The present analyses used the following 2 subscales: FR (e.g., “My child’s always asking for food” or “Even if my child is full up s/he finds room to eat his/her favourite food”) and SR (e.g., “My child gets full up easily” or “My child cannot eat a meal if s/he has had a snack just before”). All items were scored on a 5-point Likert scale as never, rarely, sometimes, often, or always. Mean scores were calculated (range: 1–5) if ≥65% of items were completed. SR and FR scales have good test-retest reliability (*r *values of 0.85 and 0.83, respectively) and high internal consistency (Cronbach’s α = 0.83 and 0.82, respectively) (8).

#### Meal variables

Diet diaries were mailed to families along with detailed instructions and portion guides, which were adapted from the preschool food atlas (24), on how to accurately estimate and record all food and drink consumed by each twin for 3 d (any 2 weekdays and 1 weekend day). Diaries were checked, coded, and linked with British food-composition tables (25) with the use of Diet In Nutrients Out, which is an in-house program developed at the Medical Research Council Human Nutrition Research in Cambridge (26).

Meals were defined as eating occasions [occasions in which food was consumed at a unique clock time (to the nearest minute on each day) including drinks consumed at the same time regardless of the amount or type of food items reported or time of day]. Occasions in which only drinks were consumed were excluded. Meal frequency was defined as the number of eating occasions per day, and meal size was defined as the amount of energy (kJ) consumed per eating occasions (total energy consumed in eating occasions divided by the total meal frequency), both of which were averaged over 3 d.

#### Demographic information

Parents reported the sex, date of birth, birth weight, ethnicity, maternal education, and gestational age (the latter 2 variables were obtained from parent-held health professional records) at age 8 mo. Ethnicity was dichotomized into white (96%) and non-white (4%) with the use of a combination of parental ethnicity. Maternal educational attainment was dichotomized into lower (no university level education) and higher (university education). Birth-weight SD scores (SDSs) were calculated for each child. Weight SDSs referenced the child’s weight against the population mean in 1990 for the child’s exact age at the time of measurement, sex, and gestational age (27). For the reference population, the mean SDS was 0 and the SD was 1; a weight SDS >0 indicated higher weight, and a weight SDS <0 indicated lower weight, compared with those of reference children of the same age, sex, and gestational age. Parents were asked to weigh and measure their children every 3 mo from birth and to use the 24-mo weight or, if this was missing, the next available weight up to 27 mo of age or the previously available weight after 21 mo of age. We computed the weight SDS, weight (kg), and weight status (overweight and normal weight) at 24 mo of age. Children were classified as overweight (*n* = 323) or normal weight (*n* = 1588) relative to the United Kingdom population mean in 1990 for the child’s age, sex, and gestational age (27). Overweight was classified as a weight SDS >1.04, which equated to a score above the 85th percentile (27), and normal weight (*n* = 1606) was classified as a weight SDS ≤1.04.

### Statistical analyses

We excluded cases with <3 d of diary entries (*n* = 378) and missing CEBQ (*n* = 118), gestational age (*n* = 25), or birth-weight (*n* = 45) data. Differences in demographic characteristics between the analysis sample (*n* = 2203) and nonresponders (*n* = 2601) were examined with the use of chi-square and independent samples *t* tests.

The correlation between FR and SR and also between meal size and meal frequency within the sample was assessed with the use of Pearson correlations. Relations between appetite (SR and FR) and meal variables (meal size and frequency) were analyzed with the use of complex samples general linear models (CSGLMs) in the SPSS version 21.0 program (SPSS Inc.); CSGLMs account for the clustering of the twins in families. Models were run with each appetitive trait as a continuous, independent variable and with each meal variable as a continuous dependent variable. Models were unadjusted and adjusted for child gestational age, birth weight, and sex as well as the difference in age at diet-diary completion and appetite measurement because these were associated with both appetite and meal variables. To take into account the possibility that heavier children may consume larger meals or eat more frequently because of higher energy needs, we also ran models with additional adjustment for previous growth (weight at 12 mo of age). Meal size and meal frequency were correlated (*r* = −0.56; *P* < 0.001), and thus, to explore the independence of their associations with SR and FR, respectively, additional models were mutually adjusted for both meal variables.

## RESULTS

Characteristics of the analysis sample (*n* = 2203) are shown in **Table 1**. On average, children were 16 mo old at CEBQ completion and 21 mo old at diet-diary completion. Compared with nonresponders, there was a slight overrepresentation of children who were younger at the CEBQ and diet-diary completion and whose mothers were of white ethnicity and educated to a higher level (*P* < 0.001).

**TABLE 1:** Characteristics of the analysis sample^1^

	Analysis sample (*n* = 2203)	Nonresponders (*n* = 2601)	*P*-between-group differences
Sex, *n* (%)			0.35^2^
Boys	1078 (48.9)	1308 (50.3)	
Girls	1125 (51.1)	1293 (49.7)	
Ethnicity, *n* (%)			<0.001^2^
White	2104 (95.5)	2362 (90.8)	
Nonwhite	99 (4.5)	229 (8.8)	
Maternal education, *n* (%)			<0.001^2^
Low	1105 (50.2)	1687 (64.9)	
High	1098 (49.8)	914 (35.1)	
Age at appetite measurement, mo	15.73 ± 1.08^3^	15.95 ± 1.21^4^	<0.001^5^
Age at diet-diary completion, mo	20.65 ± 1.10	20.96 ± 1.35^6^	<0.001^5^
Birth-weight SDS	−0.55 ± 0.93	−0.56 ± 0.96^7^	0.65^5^
Gestational age, wk	36.20 ± 2.46	36.20 ± 2.50^8^	0.98^5^
Meal frequency, times/d	4.95 ± 1.02	4.99 ± 1.20^6^	0.44^5^
Meal size, kJ	753 ± 209	724 ± 209^6^	0.006^5^
Food responsiveness (score range: 1–5)	2.22 ± 0.73	2.35 ± 0.80^4^	<0.001^5^
Satiety responsiveness (score range: 1–5)	2.68 ± 0.62	2.69 ± 0.63^4^	0.42^5^
Body weight at 24 mo of age, kg	12.30 ± 1.44	12.35 ± 1.58^9^	0.46^5^
Weight SDS at 24 mo of age	0.07 ± 1.02	0.07 ± 1.11^9^	0.95^5^
Weight status at 24 mo of age,^10^ *n* (%)			0.75^2^
Overweight	323 (16.9)	166 (17.4)	
Normal weight	1588 (83.1)	787 (82.6)	

1 *n* = 1102 families, *n* = 2203 children. SDS, SD score.

2 Chi-square test was used for differences between populations.

3 Mean ± SD (all such values).

4 *n* = 1659.

5 Independent samples *t* test was used for mean differences between populations.

6 *n* = 511.

7 *n* = 2436.

8 *n* = 2581.

9 *n* = 2581.

10 Weight status at 24 mo of age was derived with the use of weight SDSs. Children were classified as overweight (*n* = 323) or normal weight (*n* = 1588) relative to the United Kingdom population mean in 1990 for the child age, sex, and gestational age (27). Overweight was classified as a weight SDS >1.04, which equated to scores above the 85th percentile (27), and normal weight (*n* = 1606) was classified as a weight SDS ≤1.04.

The average meal frequency was 5 and ranged from 1 to 10 times/d. The average meal size was 753 kJ and ranged from 247 to 1745 kJ/meal. Meal size and meal frequency were negatively correlated (*r* = −0.56, *P* < 0.001) such that the consumption of more energy per meal was moderately associated with eating less frequently throughout the day. There was also a significant negative association between FR and SR, (*r* = −0.41, *P* < 0.001), suggesting that children who scored low on SR tended to also score high on FR.

**Table 2** presents the results of separate CSGLMs that examined associations between each appetitive trait (SR and FR) and each meal variable (meal size and frequency) unadjusted (model 1) and adjusted for covariates (models 2 and 3). In all models, as predicted, SR was significantly and negatively associated with meal size. A one-unit increase in the SR scale was associated with children consuming ∼50 kJ less per meal. A child who scored 5 on the SR scale (most satiety responsive) would consume, on average, 200 kJ less at each meal than a child would who scored one (least satiety responsive). On the basis of an average of 5 meals/d, this difference could equate to 1000 kJ less per day. Also in line with our predictions, in all models, FR was significantly associated with meal frequency with more food-responsive children eating more frequently over the course of a day. The change in meal frequency for a one-unit change in FR was 0.11; a child who scored 5 on the FR scale (most food responsive) would eat ∼0.5 meals/d more than a child would who scored one (least food responsive). On the basis of the average meal size of 753 kJ, this could equate to 331 kJ more per day. SR was not associated with meal frequency, and FR was not associated with meal size. All associations remained when adjusted for child sex, gestational age, birth weight, the difference in age between diary completion and CEBQ completion, previous growth, and mutual adjustment for each meal variable, which provided support for independent effects of SR on meal size and FR on meal frequency (Table 2, model 3).

**TABLE 2:** Associations between appetitive traits (satiety responsiveness and food responsiveness) and meal variables (meal size and meal frequency)^1^

	Appetitive trait			
	Satiety responsiveness		Food responsiveness	
Meal variable and model	*B*^2^ ± SE	*P*^3^	*B* ± SE	*P*^4^
Meal size, kJ				
Model 1	−51.76 ± 8.74	<0.001^5^	−2.64 ± 8.03	0.74
Model 2	−47.61 ± 8.79	<0.001^5^	−6.53 ± 7.91	0.41
Model 3	−39.29 ± 7.57	<0.001^5^	8.49 ± 6.82	0.21
Meal frequency, times/d				
Model 1	0.06 ± 0.04	0.19	0.13 ± 0.04	0.001^5^
Model 2	0.06 ± 0.04	0.15	0.13 ± 0.04	0.001^5^
Model 3	−0.07 ± 0.04	0.07	0.11 ± 0.03	0.001^5^

1 Models are complex samples general linear regression models. Model 1 was adjusted for the twin structure of the dataset and unadjusted for covariates. Model 2 was adjusted for the twin structure of the dataset and the covariates sex, gestational age, birth weight, difference in age between diet-diary completion, and Child Eating Behavior Questionnaire completion. Model 3 was adjusted for the covariates sex, gestational age, birth weight, difference in age between diet-diary completion, and Child Eating Behavior Questionnaire completion and also mutually adjusted for meal size and meal frequency to allow for the assessment of independent effects of appetitive traits on meal variables. For model 3, results were unchanged with additional adjustment for previous growth (weight at 12 mo of age). *n* = 2203.

2 *B*, unstandardized β coefficient.

3 All *P* values are for the significance of the *B *coefficient for associations between SR and meal variables.

4 All* P *values are for the significance of the *B* coefficient for associations between FR and meal variables.

5 *P* < 0.05.

## DISCUSSION

### Summary of findings

To our knowledge, this is the first study to explore associations between appetitive traits and young children’s eating patterns in the home context, which is crucial for understanding how to design practical and targeted interventions to prevent excessive weight gain in children who are behaviorally susceptible to obesity. As hypothesized, the results show that children with higher FR eat more frequently without any average difference in meal size, whereas children with lower SR consume more energy per meal without any average difference in meal frequency.

These findings help us to understand the behavioral expression of the appetitive traits that have been linked with risk of weight gain. There is a plethora of research that has shown that children with low SR and high FR tend to be heavier (1, 13, 14, 16, 17, 28, 29) including research that used the current sample (15, 18). Comprehensive dietary data indicate that meal size and meal frequency may be potential mechanisms through which children with low SR or high FR are at risk of weight gain; food-responsive children eat more frequently, and children with lower SR consume more energy each time that they eat.

Previous studies have suggested that toddlers and infants self-regulate their energy intake by adjusting their portion sizes depending on the number of eating occasions in a given day (30, 31). In a study of 4–24-mo0old children, Fox et al. (31) showed that children who ate less often during the day consumed larger-than-average portion sizes, and children who ate more often during the day consumed smaller-than-average portions (31). In the current study, there was a significant negative association between meal size and meal frequency within the sample as a whole, which suggested that children regulate their energy intakes; however, it appears that there may be individual differences in this self-regulation ability because children who scored low on SR were also likely to score high on FR. We have shown independent effects of each appetitive trait on each meal variable, which suggested that children with high FR appear not to compensate for more-frequent eating by consuming less energy per eating occasion because FR is associated with meal frequency when meal size is held constant, and FR is not associated with meal size. Similarly, children with poor SR appear not to compensate for larger meal sizes by eating less frequently because SR is associated with meal size when meal frequency is held constant, and SR is not associated with meal frequency. These findings indicate that children who exhibit these appetitive characteristics may be poorer at energy self-regulation and, therefore, more susceptible to weight gain. A portion-size intervention may be necessary for children with poor SR, whereas a food-responsive child may require an alternative intervention to prevent excessive weight gain, e.g., by reducing the number of eating occasions per day.

### Strengths and limitations

The Gemini study is a large national cohort, with detailed measures of diet, enabling meal variables to be characterized. Diet diaries are considered an accurate method of dietary data collection (32, 33), and although they are prone to reporting error, parents were provided with detailed portion guides to minimize this error and ensure the standardization of dietary reporting (34).

The predominantly white, highly educated sample may have created bias; however adjustment was made for this possibility as well as a broad range of other potentially confounding factors. The twin nature of the sample questions how generalizable the findings are to a population of singletons; however, Mallan et al.(28) reported, in a sample of 2-y-old singletons, similar mean scores for FR (mean: 2.19 compared with 2.22) and SR (mean: 2.97 compared with 2.68), and dietary data from 386 singletons aged 1–3 y old in the National Diet and Nutrition Survey (http://discover.ukdataservice.ac.uk/catalogue?sn=6533) are also comparable with an average meal frequency of 5.3 and an average meal size of 833kJ.

The results of this study cannot be generalized beyond the relatively young age of the sample, and it is possible that the relations between appetitive traits and eating patterns change as children get older. For example, early in life, eating frequency may largely be under the control of parents, with toddlers having very little free choice over how often they eat. In contrast, the amount consumed at each sitting may be more within the control of children, e.g., by finishing everything on the plate or leaving it as they wish. Future work should explore appetitive traits and eating patterns at older ages when children have more autonomy with respect to how often and how much they consume as well as the role of other potentially pervasive influences on eating patterns including parental BMI, socioeconomic status, and education, which may play a role.

In conclusion, the pathway between the 2 key appetitive traits FR and SR and eating patterns appears to be different. These idiosyncratic traits each have the potential to tip a child into a positive energy balance. The assessment of appetitive traits in early childhood could help to identify individuals with high-risk appetitive characteristics. More guidance could be offered to parents on the recommended eating frequency and portion sizes for infants and toddlers on the basis of each child’s respective appetitive traits.

## Footnotes

4 Abbreviations used: CEBQ, Child Eating Behavior Questionnaire; CSGLM, complex samples general linear model; FR, food responsiveness; SDS, SD score; SR, satiety responsiveness.
